# Antiseptic Therapeutics1Presented before the meeting of the American Veterinary Medical Association, Detroit, Mich., September, 1900.

**Published:** 1900-12

**Authors:** J. F. Winchester

**Affiliations:** Lawrence, Mass.


### ANTISEPTIC THERAPEUTICS.[1]

By J. F. Winchester, B.Sc., D.V.S.,

LAWRENCE, MASS.

(Concluded from page 654.)

Iodine is an active antiseptic whether used in the gaseous, liquid, or solid state. One part in 4125 parts of water arrests the action of diastase and ptyalin; one part in 7817 arrests the action of pepsin; one part in 7000 destroys both bacilli and their spores (Wernitz and Koch). Structures, whether natural or morbid, may be gradually liquefied and absorbed by its use. Medicinal doses are absorbed; they also stimulate glandular activity and promote metabolism. In the tissues iodine may again be set free and combine with serum albumin, but iodine albuminates are unstable, and hence readily removed.

This appears to explain the action of iodine and its compounds in the liquefaction and absorption of pathological products. It is excreted by the mucous surfaces and glands, being found notably in the saliva, perspiration, and urine.

Dr. H. L. Jenckes, in his notes on the "Therapeutics of Croupous Pneumonia," refers to a remedy which has been advocated as having a destructive or at least an inhibitory action on the pneumococcus. This is ethyl iodide. He quotes Professor Bartholow, who says: "The influence exerted by it on the cough, the labored breathing, and the general malaise is a remark-

[1] Presented before the meeting of the American Veterinary Medical Association, Detroit, Mich., September, 1900.

able fact. It has the single advantage that no apparatus is required; it vaporizes at ordinary temperature; it causes no irritation of the mucous membrane, and it does not act as an anæsthetic. The cases in which it was used were all apparently much benefited by its inhalation, and all expressed a sense of relief to the general malaise and to the specific symptoms—cough, bronchial irritation, and expectoration—the last mentioned being soon changed in character and in quantity."

Ethyl iodide should be freely inhaled, not only for its supposed specific effect upon the pneumococcus, but also for the relief it affords the pulmonary symptoms. Compressed air and creosote are recommended by Professor See in apical and fetid bronchitis, the inhalation to be made daily or more frequently. The results obtained would appear to be surprising, a marked amelioration being observable in most cases. Professor W. L. Williams says that intratracheal medication has much to recommend it when rapid effects are desired, especially in those pulmonary diseases where antiseptics are indicated. At the time of writing his article on the subject he had not met with noteworthy success in treating suppurative bronchitis by intratracheal injections of antiseptics, and he was unable to find any record of attempts to administer per trachea for therapeutic purposes large volumes of liquids either as mechanical detergents or as topical or general antiseptics.

By referring to his article in the *Veterinary Review*, vol. xxi., No. 9, you will find a very interesting account of two cases of traumatic bronchitis treated antiseptically through the trachea.

Dr. Jules Daurial gives his observation of lavage of the whole intestinal tract in eleven infants who were suffering from obstinate fetid diarrhœa, which checked the disease in all instances. The fluid used was weak solutions of lactic acid and creolin. One case of typhoid fever was treated in the same way, with good results. Dr. W. W. Johnson, of Washington, advocates the local antiseptic treatment of acute dysentery, from the fact that the affection itself was local, being confined to the colon and the rectum.

The rationale of the method was shown in the fact that we had a local inflammatory process with rapid infiltration of tissue; a tendency to ulceration and necrosis and extensive destruction; while the decomposition and retention of decomposing solids and fluids in the bowels would result in auto-infection, with also the probable dependence of these conditions upon the introduction of a parasite.

In closing this paper I shall digress from my first statement, that new material would not be introduced, and will call your attention to some facts by Dr. A. W. Balch and Mr. W. R. Brinckerhoff, of the Harvard Medical School, relating to a "Possible Cause of Azoturia" and the "Pathology of Azoturia."

